# Supplementary material for: Analysis of PPARγ Signaling Activity in Psoriasis
Source: Int J Mol Sci. 2021 Aug 10;22(16):8603. doi: 10.3390/ijms22168603 (PMC8395241; doi:10.3390/ijms22168603)
Supplement: Supplementary file 1 [file ijms-22-08603-s001.zip › Supplemental materials_Analysis of PPARg signaling activity in psoriasis/Pathway models/Models images and html files/Interleukin-17 and interleukin-22 signaling in psoriasis/8671.html]

PDPK1


# Protein PDPK1

|  |  |
| --- | --- |
| URN | urn:agi-llid:5170 |
| Total Entities | 0 |
| Connectivity | 2441 |
| Name | PDPK1 |
| Description | 3-phosphoinositide dependent protein kinase 1 |
| Notes | may catalyze the phosphorylation and activation of protein kinase B [RGD, Feb 2006] |
| Primary Cell Localization | Cytoplasm |
| Class | Protein kinase |
| ObjectType | Protein |

---

|  |  |
| --- | --- |
| Pathway | Osteoarthritis |
|  | Oncogene Induced Cellular Senescence |
|  | MTHFR Activity Regulation |
|  | DNA Persistent Repair Inhibits mTOR Signaling |
|  | mTOR Signaling Activation by Fatty Acids and Glucose |
|  | mTOR Signaling Activation by Amino Acids |
|  | mTOR Signaling |
|  | mTOR Signaling Hyperfunction |
|  | Insulin Influence on Protein Synthesis |
|  | Insulin Influence on Glycogenesis |
|  | Insulin Influence on Lipogenesis |
|  | Dopamine Receptors Signaling |
|  | Serotonin Receptors Signaling |
|  | Thyroid Hormones Common non-Genomic Effects in Hyperthyroidism |
|  | Insulin Signaling |
|  | Thrombopoietin Receptors Signaling in Platelet Maturation |
|  | LH and FSH Signaling and Steroidogenesis |
|  | Toll-like Receptors Act through MYD88-TIRAP Signaling |
|  | T-Cell Dependent B-Cell Activation |
|  | T-Cell Independent B-Cell Activation |
|  | Natural Killer Cell Activation through C-Type Lectin-like Receptors |
|  | Natural Killer Cell Activation through ITAM-Containing Receptors |
|  | Natural Killer Cell Activation through ITSM-Containing Receptors |
|  | T-Cell Maturation (Hypothesis) |
|  | Peripheral T-Cell Tolerance |
|  | T-Cell Positive Selection and Neglect Induced Death |
|  | T-Cell Central Tolerance |
|  | CD8+ T-Cell Activation |
|  | Th1-Cell Differentiation |
|  | Treg-Cell Differentiation |
|  | Th2-Cell Differentiation |
|  | T-Cell Receptor Signaling |
|  | Th17-Cell Differentiation |
|  | AHR Signaling in Tr1 Cells Function |
|  | CXC Chemokine Receptor Signaling |
|  | CC Chemokine Receptor Signaling |
|  | Plasmin Effects in Inflammation |
|  | TAM Receptors in Platelet Aggregation |
|  | Platelet Activation via Adhesion Molecules |
|  | Scavenger Receptors in Platelet Aggregation |
|  | Proplatelet Maturation |
|  | Bradykinin Effects in Inflammation |
|  | Platelet Activation via GPCR Signaling |
|  | Toll-like Receptors in Sterile Inflammation |
|  | Toll-like Receptor Independent Sterile Inflammation |
|  | Lipoxin A4/FPR2-Related Neutrophil Depression |
|  | Eicosanoids in Myeloid Cell Activation |
|  | Histamine Receptor H3 Signaling in Neurons |
|  | Neutrophil Activation via LTB4/Polyisoprenyl Phosphate Signaling |
|  | Basophil Activation |
|  | Eosinophil Survival by Cytokine Signaling |
|  | Eosinophil Activation |
|  | Mast-Cell Activation via IgE Signaling |
|  | Mast-Cell Activation without Degranulation through CYSLTR1/CYSLTR2 Signaling |
|  | Mast-Cell Activation without Degranulation |
|  | Mast-Cell Activation without Degranulation through KIT/F2R Signaling |
|  | Mast-Cells De Novo Synthesized Mediators via IgE Independent Signaling |
|  | CD16/CD14 Proinflammatory Monocyte Activation |
|  | Neutrophil Activation via FCGR3B |
|  | Neutrophil Degranulation via FPR1 Signaling |
|  | Neutrophil Chemotaxis |
|  | Neutrophil Activation via Adherence on Endothelial Cells |
|  | VEGFA/NOTCH1/WNT Cross-talk in Blood Vessel Sprouting and Branching in Cancer |
|  | Vascular Endothelial Cell Activation by Cytokines |
|  | Vascular Endothelial Cell Activation by Blood Coagulation Factors |
|  | Vascular Endothelial Cell Activation by Growth Factors |
|  | Vascular Endothelial Cell Permeability Activation |
|  | Lipogenesis Regulation in Adipocyte |
|  | Synaptic Potentiation by PKMZ in LTP Maintenance - Active State |
|  | Potassium Flux Regulation |
|  | GFs/TNF -> Ion Channels |
|  | Nociception Expression Targets Signaling |
|  | Sodium Flux Regulation |
|  | Vascular Motility |
|  | Ca2+ Flux Regulation |
|  | NPY1R -> CRH/POMC Production |
|  | NTSR1 Expression Targets |
|  | BDKRB1/2 -> Vasodilation |
|  | BDKRB1/2 -> Interleukins Production |
|  | CNR1/2 -> Membrane Transport |
|  | CNR1/2 -> IL1B/2/4/6/10 Production |
|  | DRD2 Expression Targets |
|  | DRD2 -> TRPC1 Transcription |
|  | DRD2/4 -> Membrane Transport |
|  | GALR1/2/3 -> POMC/NPY Production |
|  | CHRM1 -> IL2 Production |
|  | CHRM1/2/3/5 -> Ion Channels |
|  | PTGER2/3 -> Vascular Motility |
|  | HTR1 -> IL6 Production |
|  | HTR5 -> TNF Production |
|  | HTR1 -> Membrane Transport |
|  | TACR1 -> TNF/IL6/IL8 Production |
|  | Leptin -> NO Production/Vasodilation |
|  | NTRK1/2/3 -> Acetylcholine Production |
|  | T-lymphoid precursor cell -> CD8+ Naive T-cell Surface Expression Markers |
|  | G0/G1 Phase Transition |
|  | RAGE/AGER and S100 Proteins in Cardiovascular Injury |
|  | Th1-Cells Activation and Proliferation in Atherosclerosis |
|  | Dilated Cardiomyopathy |
|  | Endothelin Signaling in Arterial Hypertension |
|  | Arterial Hypertension |
|  | Endothelial Cell Dysfunction in Arterial Hypertension |
|  | Smooth Muscle Cell Dysfunction in Arterial Hypertension |
|  | TRPM4/6/7/8 Signaling in Arterial Hypertension (Hypothesis) |
|  | Smooth Muscle Cell Dysfunction in Pulmonary Hypertension |
|  | Myocardial Remodeling |
|  | Myocardial Infarction |
|  | Proteins Involved in Myocardial Ischemia |
|  | Preconditioning Ischemia |
|  | Lymphocyte Mediated Myocardial Injury in Myocarditis |
|  | Myocarditis, Acute Viral Overiew |
|  | Vascular Reactivity in Raynaud Disease |
|  | NO Synthesis in Early Vasospasm |
|  | Neurons Necrosis Caused by Energy Deficiency |
|  | Contraction Due Vasospasm |
|  | Hyperseborrhea in Acne Vulgaris |
|  | Androgen Induced Sebocyte Hyperfunction |
|  | Androgens in Sebocyte Maturation |
|  | Atopic Dermatitis Onset |
|  | Cell Cycle Dysregulation in Melanoma |
|  | Melanoma |
|  | Apoptosis Block in Melanoma Cell |
|  | Dedifferentiation and Metastatic Progression in Melanoma |
|  | MITF as a Regulator of Melanoma Cell Development |
|  | Proteins Involved in Melanoma |
|  | IFN-gamma/TNF-alpha Mediated Cell Proliferation in Psoriasis |
|  | IL22 Induces Keratinocyte Proliferation in Psoriasis |
|  | Dendritic Cells Function in Psoriasis |
|  | Th0-Cell Aberrant Activation in Systemic Lupus Erythematosus |
|  | Th1-Cell Function in Systemic Lupus Erythematosus |
|  | B-Cell Activation in Systemic Lupus Erythematosus |
|  | Th2-Cell Function in Systemic Lupus Erythematosus |
|  | Acute Cytotoxic CD8+T-Cell Response against Melanocytes |
|  | Vitiligo |
|  | T-Cell Cytotoxic Response against Melanocytes in Vitiligo |
|  | Apoptosis of Melanocyte in Vitiligo |
|  | Humoral Immunity in Vitiligo |
|  | Treg-Cell Function in Diabetes Mellitus Type 1 |
|  | Natural Killer T-Cell Roles in Diabetes Mellitus Type 1 |
|  | Toll-like Receptors in beta-Cell |
|  | Macrophage Function in Diabetes Mellitus Type 1 |
|  | Treg-Cell Activation in Diabetes Mellitus |
|  | Donohue Syndrome Progression (Hypothesis) |
|  | Advanced Glycation End Products Pathway in Diabetic Microangiopathy |
|  | Peripheral Tissue Microangiopathy |
|  | Tubular Cell Dysfunction in Progressive Diabetic Nephropathy |
|  | Podocyte Dysfunction in Diabetic Nephropathy |
|  | Endothelial Cell Dysfunction in Early Diabetic Nephropathy |
|  | Mesangial Cell Dysfunction in Diabetic Nephropathy |
|  | Microangiopathy in Diabetic Neuropathy |
|  | Proliferative Diabetic Retinopathy |
|  | Adipokines and Cytokines in Insulin Resistance in Skeletal Muscles |
|  | AGE Related Insulin Resistance in Skeletal Muscles |
|  | Gluconeogenesis Impairment in non-Alcoholic Fatty Liver Disease |
|  | PNPLA3 in non-Alcoholic Fatty Liver Disease |
|  | Lipid Metabolism Impairement in non-Alcoholic Fatty Liver Disease |
|  | Leptin in Insulin Synthesis and Secretion |
|  | beta-Cell Destruction through Cytokines in Diabetes Mellitus |
|  | beta-Cell Mass Regulation |
|  | beta-Cell Function Inhibition by Ciclosporine and Tacrolimus (Rodent Model) |
|  | FOXO1 and SREBP-1C Role in beta-Cell Suppression (Rodent Model) |
|  | RET1 Mutation in Hyperparathyroidism |
|  | Bone Remodeling in Hyperthyroidism |
|  | Cardiovascular Effects in Hyperthyroidism |
|  | Thyroid Dysfunction |
|  | Graves Ophthalmopathy |
|  | Immune System Activation in Hyperthyroidism |
|  | Hypothyroidism, Secondary (Central) |
|  | Thyroid Stimulating Hormone (TSH) Resistance in Congenital Hypothyroidism |
|  | Overt Hypothyroidism, Primary |
|  | Thyroid Stimulating Hormone (TSH) Secretion in Overt Hypothyroidism |
|  | Thymic Follicular Hyperplasia |
|  | Adipocyte Hypertrophy and Hyperplasia |
|  | CD4+T-Cell Response in Celiac Disease |
|  | Enterocyte Mediated Mucosal Damage in Celiac Disease |
|  | Metastatic Colorectal Cancer |
|  | GUCY2C Signaling in Colorectal Cancer |
|  | OipA Signaling in Helicobacter Pylori Infection |
|  | Peutz-Jeghers Syndrome |
|  | Dendritic Cell Function in Ulcerative Colitis |
|  | Nicotine anti-Inflammatory Effect in Ulcerative Colitis |
|  | Th2-Cell Function in Ulcerative Colitis |
|  | T-Cell Cytotoxic Response in Ulcerative Colitis |
|  | FLT3/KIT/MLL Signaling in Leukemia |
|  | Apoptosis Block in Acute Myeloid Leukemia Cell |
|  | RARA Signaling in Acute Myeloid Leukemia (M3) |
|  | Acute Myeloid Leukemia |
|  | Antiphospholipid Antibodies in Platelets and Coagulation Cascade |
|  | B-Cell Acute Lymphoblastic Leukemia |
|  | B-Cell Chronic Lymphocytic Leukemia |
|  | Chronic Myeloid Leukemia |
|  | Diffuse Large-B-Cell Lymphoma |
|  | Diffuse Large-B-Cell Lymphoma ABC Subtype |
|  | Lymphoma, GCB Subtype |
|  | Follicular Lymphoma |
|  | Hodgkin Lymphoma |
|  | Mantle Cell Lymphoma |
|  | mTOR/NF-kB/BCR Signaling Disregulation in Mantle Cell Lymphoma |
|  | Hedgehog Signaling in Mantle Cell Lymphoma |
|  | Mucosa-Associated Lymphoid Tissue (MALT) Lymphoma |
|  | Osteoclast Activation in Multiple Myeloma |
|  | IL6/IGF1/VEGFA Signaling in Multiple Myeloma |
|  | Multiple Myeloma |
|  | Platelet and Coagulation Cascade Activation by Antiphospholipid Antibodies |
|  | T-Cell Acute Lymphoblastic Leukemia |
|  | Hepatocellular Carcinoma |
|  | Growth Factor Signaling in Hepatocellular Carcinoma |
|  | Pancreatic Ductal Carcinoma |
|  | Growth Factor Signaling in Pancreatic Neoplasms |
|  | Pancreatic Neuroendocrine Tumors |
|  | STK11 Signaling in Pancreatic Neoplasms |
|  | Il17 Signaling Related Neutrophilia in Asthma |
|  | Airway Smooth Muscle Cell High Rate Proliferation |
|  | IgE Induces Airway Smooth Muscle Cell Proliferation |
|  | Goblet-Cell Exocytosis in Asthma |
|  | Goblet-Cell Related Mucus Secretion in Asthma |
|  | Neutrophil Degranulation via FPR1/IL8 |
|  | Basophil Activation in Asthma |
|  | Mast-Cell Activation in Asthma |
|  | Mast-Cell Related Chemokines Signaling in Asthma |
|  | B-Cell Mediated IgE Production in Asthma |
|  | Th17-Cell Differentiation in Asthma |
|  | Th2-Cell Response in Asthma |
|  | Ovulation Block |
|  | SHBG and IGFBP1 Synthesis Decline in Polycystic Ovary Syndrome |
|  | Polycystic Ovary Syndrome |
|  | FSH Action in Polycystic Ovary Syndrome |
|  | Dendritic Cell Dysfunction in Psoriatic Arthritis |
|  | Th17-Cell and Th1 Immune Responsein Psoriatic Arthritis |
|  | Keratinocyte Activation in Psoriatic Arthritis |
|  | B-Cells Function in Systemic Scleroderma |
|  | Th2-Cells Function in Systemic Scleroderma |
|  | CD4+ T-Cell Function Decline in HIV |
|  | Macrophage Survival through CCR5 and CXCR4 Mediated Signaling |
|  | Toll-like Receptors (TLR) in Periodontitis |
|  | Diabetes Induced Periodontitis |
|  | Estrogen Effects on Pregnancy Gingivitis |
|  | IL1 Receptor Antagonist (IL1RN) Deficiency (DIRA) |
|  | Mevalonate Kinase Deficiency |
|  | Blau Syndrome |
|  | Familial Cold Autoinflammatory Syndrome 2 |
|  | Hereditary Hemorrhagic Telangiectasia |
|  | Cowden Syndrome |
|  | Glutamate Toxiicity Effects on Striatum Medium Spiny Neurons in Hantington's Disease |
|  | IL6 and Insulin Resistance in Muscular Dystrophies |
|  | Ca2+ Overload in Muscular Dystrophies |
|  | Muscular Dystrophies |
|  | Dystrophin Glycoprotein Complex Signaling in Muscular Dystrophies |
|  | IGF1 Role in Muscle Hypertrophy |
|  | Myostatin-IGF1 Crosstalk in Skeletal Muscles in Muscular Dystrophies |
|  | Osteoblast Function Decline in Osteoporosis |
|  | BDNF Upregulation Triggered by Seizures |
|  | Ketogenic Diet Mechanism (Hypothesis) |
|  | Blood-Brain Barrier Disruption in Epileptiform Disorders |
|  | mTOR Hyperactivation after Status Epilepticus |
|  | Glioblastoma, Primary |
|  | Glioblastoma, Secondary |
|  | Astrocytoma |
|  | Glioma Invasion Signaling |
|  | Glioma Stem Cell Program Activation |
|  | Ependymoma |
|  | Medulloblastoma |
|  | Vascular Endothelial Cell Vasodilation in Brain |
|  | Dopamine Action in CSD Progression |
|  | Postsynaptic Neuron Activation |
|  | nNOS (NOS1) and iNOS (NOS2) Activation via Glutamate |
|  | Growth Factor Signaling in Neuroblastoma |
|  | Neuroblastoma |
|  | ALK Associated Neuroblastoma |
|  | Glutamate Role in Parkinson's Disease |
|  | Glutamate Mediated Excitotoxicity in Amyotrophic Lateral Sclerosis |
|  | Microglia and Motor Neuron Interaction Dysregulation |
|  | MIR155 Role in Th17-Cell in Multiple Sclerosis |
|  | Brain Cell Necrosis in Vascular Dementia |
|  | Proteins Involved in Breast Cancer Related to ESR1 Signaling Pathway |
|  | Proteins Involved in Breast Cancer Related to ERBB2/VEGFR/Akt Signaling Pathway |
|  | ERBB/VEGFR/Akt Signaling in Breast Cancer |
|  | IGF1R/AKT Signaling in Breast Cancer |
|  | ESR1/ERBB Positive Luminal Breast Cancer |
|  | Proteins Involved in Breast Cancer Related to IGF1R/Akt Signaling Pathway |
|  | Breast Cancer |
|  | ESR1 Signaling in Breast Cancer |
|  | Endometrial Cancer |
|  | Endometrioid Endometrial Cancer |
|  | Clear Cell Endometrial Cancer and Papillary Serous Endometrial Cancer |
|  | Angiogenesis in Endometriosis |
|  | Coagulation Factors and PAR1/2 Receptors (F2R/F2RL1) in Endometriosis |
|  | Endometrioid Ovarian Carcinoma |
|  | Clear Cell Ovarian Carcinoma |
|  | High-Grade Serous Ovarian Carcinoma |
|  | Low-Grade Serous Ovarian Carcinoma |
|  | Ovarian Cancer |
|  | Mucinous Ovarian Carcinoma |
|  | LH and FSH Signaling Impairment in Primary Ovarian Insufficiency |
|  | FOXO3 and PTEN Inactivation in Premature Ovarian Failure (Mouse Model) |
|  | MIF Role in Cholesteatoma |
|  | EGF/cyclinD1 Signaling in Acquired Middle Ear Cholesteatoma |
|  | HMGB1/RAGE Signaling in Middle Ear Cholesteatoma |
|  | Cholesteatoma |
|  | HPV E5 Regulates EDNRA and EGFR Signaling Pathway in Keratinocytes |
|  | Androgen Receptor/Akt Signaling |
|  | Prostate Cancer |
|  | Androgen Receptor/SGK1 Signaling |
|  | Steroids Induced Cataract |
|  | VEGFA in Neovascular Glaucoma |
|  | Glutamate Mediated Excitotoxicity in Glaucoma |
|  | Neurotrophic Factor Deprivation in Retinal Ganglion Cell Death |
|  | Immune System Role in Otitis Media |
|  | Alveolar Macrophages Dysfunction in COPD |
|  | T-Cell Cytotoxic Activation |
|  | Alveolar Epithelial Cell Dysfunction in COPD |
|  | Polycystins Mutation Effects in Cystic Kidney Disease |
|  | Penile Detumescense Augmentation Mechanisms |
|  | Advanced Glycation End Products in Diabetes or Age Related Erectile Dysfunction |
|  | Plasmin Role in Glomerulonephritis |
|  | Endothelial Cell Dysfunction in Glomerulonephritis |
|  | Ammonia Effects on Brain Cells |
|  | CD157/ITGB2 Signaling in Myeloid Cell |
|  | CD38/CD3 -> JUN/FOS/NF-kB Signaling in T-cell Proliferation |
|  | CD38/CD19 -> JUN/FOS/NF-kB Signaling in B-cell Proliferation |
|  | S100P Expression Target |
|  | HMGB1 Expression Targets |
|  | TTR Expression Targets |
|  | S100B Expression Targets |
|  | ANGPT1 -> CREB/CREBBP Expression Targets |
|  | ANGPT2 -> AP-1/CREBBP/MYC Expression Targets |
|  | AREG -> NFATC Expression Target |
|  | AREG -> CREB/CREBBP Expression Targets |
|  | AREG -> NCOR2 Expression Targets |
|  | AREG -> HIF1A Expression Targets |
|  | AREG -> AP-1 Expression Targets |
|  | AREG -> FOXO3A Expression Target |
|  | Noradrenaline/Gs Expression Targets |
|  | Epinephrine/Gs Expression Targets |
|  | Acetylcholine Expression Targets |
|  | Dopamine/Gi Expression Targets |
|  | Aryl Hydrocarbon Receptor Genomic and non-Genomic Signaling |
|  | BTC -> NFATC Expression Targets |
|  | BTC -> AP-1/ATF/CREB Expression Targets |
|  | BTC -> EP300/ETS/ETV/SP1 Expression Targets |
|  | AGTR1 -> ARRB1/ARRB2 Signaling |
|  | Hedgehog Family -> ARRB1/ARRB2 Canonical Signaling |
|  | CD19 Expression Targets |
|  | CD81 -> AP-1 Expression Targets |
|  | CD247 Expression Targets |
|  | FCGR3A Expression Targets |
|  | CD19/CR2 Expression Targets |
|  | Tamoxifen Induced Endometrial Cancer |
|  | Dexamethasone Induced Diabetes |
|  | Doxorubicin Induced Cardiotoxicity |
|  | Ritonavir Induced Diabetes |
|  | Clozapine Induced Granulocytopenia |
|  | EGF -> HIF1A Expression Targets |
|  | EGF -> AP-1/ATF Expression Targets |
|  | EGF -> CREB/CREBBP/ELK/SRF/MYC Expression Targets |
|  | EGF -> MEF/MYOD/NFATC Expression Targets |
|  | EGF -> FOXO3A Expression Targets |
|  | EGF -> TP53 Expression Targets |
|  | EGF -> NCOR2 Expression Target |
|  | EGFR -> NCOR2 Signaling |
|  | EGFR/ERBB3 -> MEF/MYOD/NFATC/MYOG Signaling |
|  | EGFR/ERBB2 -> TP53 Signaling |
|  | ERBB2/3 -> EP300/ETS/ETV/SP1 Signaling |
|  | EGFR/ERBB2 -> HIF1A Signaling |
|  | EGFR -> AP-1/ATF2 Signaling |
|  | EGFR -> AP-1/CREB/ELK/SRF/MYC Signaling |
|  | EREG -> CREB Expression Target |
|  | EREG -> EP300/SP1 Expression Targets |
|  | EREG -> FOXO3A Expression Target |
|  | EREG -> HIF1A Expression Target |
|  | EREG -> AP-1/ATF Expression Targets |
|  | Erythropoietin -> FOXO3A Expression Targets |
|  | Erythropoietin -> NF-kB Expression Targets |
|  | Erythropoietin -> AP-1/MYC/CREB Expression Targets |
|  | Erythropoietin -> ELK/SRF Expression Targets |
|  | PECAM1 Expression Targets |
|  | F2 -> AP-1/CREB/ELK/SRF/SP1 Expression Targets |
|  | F2 -> STAT1/NF-kB Expression Targets |
|  | PLG -> AP-1/CREB/ELK/SRF/SP1 Expression Targets |
|  | PLG -> STAT1/NF-kB Expression Targets |
|  | FGF9 -> AP-1/CREB/MYC Expression Targets |
|  | FGF10 -> AP-1/CREB/CREBBP/MYC Expression Targets |
|  | FGF2 -> FOXO3A Expression Targets |
|  | FGF1 -> AP-1/CREB/ELK/SRF/MYC Expression Targets |
|  | FGF1 -> NCOR2 Expression Target |
|  | FGF7 -> FOXO3A Expression Target |
|  | FGF8 -> AP-1/CREB/MYC Expression Targets |
|  | FGF18 -> AP-1/CREB Expression Targets |
|  | FGF23 -> NCOR2 Expression Targets |
|  | FGF10 -> FOXO3A Expression Target |
|  | FGF2 -> NCOR2 Expression Targets |
|  | FGF4 -> AP-1/MYC Expression Targets |
|  | FGF7 -> AP-1/CREB/CREBBP/MYC Expression Targets |
|  | FGF2 -> AP-1/CREB/CREBBP/ELK/SRF/MYC Expression Targets |
|  | FGFR -> AP-1/CREB/CREBBP/ELK/SRF/MYC Signaling |
|  | FSHR Expression Targets |
|  | GH1/GHR -> NF-kB/ELK/SRF/MYC Expression Targets |
|  | CSH1/GHR Expression Targets |
|  | HBEGF -> FOXO3A Expression Target |
|  | HBEGF -> AP-1/ATF Expression Targets |
|  | HBEGF -> TP53 Expression Targets |
|  | HBEGF -> EP300/ETS/ETV/SP1 Expression Targets |
|  | HBEGF -> HIF1A Expression Targets |
|  | HBEGF -> MEF/MYOD Expression Target |
|  | HBEGF -> CREB/MYC Expression Targets |
|  | HGF -> FOXO3A Expression Targets |
|  | HGF -> AP-1/CREB/ELK/SRF/MYC Expression Targets |
|  | HGFR -> FOXO3A Signaling |
|  | HGFR -> AP-1/CREB/MYC Signaling |
|  | TAM Receptor Signaling Activation in Cancer |
|  | PI3K/AKT/MTOR Signaling Activation in Cancer |
|  | G0/G1 Cell Cycle Phase Transition Activation in Cancer |
|  | RAS/RAF/MAPK Signaling Activation in Cancer |
|  | N2 Neutrophils in Tumor-Promoting Inflammation and Tumor Progression |
|  | RAS/RAF/MAPK Signaling Activation by Blocking of Tumor Suppressors |
|  | TGFB Signaling activation by Blocking of Tumor Suppressors |
|  | PI3K/AKT/MTOR Signaling Activation by Blocking of Tumor Suppressors |
|  | Hypoxia-Induced Cell Death Evasion in Cancer |
|  | HIF1A in Vasculogenic Mimicry of Cancer |
|  | VEGFA Dependent Angiogenesis in Cancer |
|  | ANGPT/TEK Maintains Endothelial Cell Quiescence in Cancer |
|  | Inhibition of THBS1 Activates Angiogenesis in Cancer |
|  | VEGF Independent Angiogenesis in Cancer |
|  | Proteins with Altered Expression in Cancer Metastases |
|  | Epithelial to Mesenchymal Transition in Cancer: Overview |
|  | MTOR/TORC in Cancer Cell Motility and Invasion |
|  | Hyaluronic Acid, CD44 and HMMR in Cancer Cell Invasion and Survival |
|  | CDH2 Activation Promotes Cancer Cell Migration and Survival |
|  | TGFB Family in Epithelial to Mesenchymal Transition in Cancer |
|  | Invadopodia Formation in Cancer Cells |
|  | Skeletal Muscle Wasting in Cancer Cachexia |
|  | Metabolic Effects of Oncogenes and Tumor Suppressor in Cancer Cells |
|  | Lipogenesis/Lipolysis Activation in Cancer Cells and Cancer-Associated Adipocytes |
|  | Treg Cells Promote Immunosuppression in Cancer Immune Escape |
|  | IDO1 in Cancer Immune Escape |
|  | Effector T-cell Inactivation in Cancer Immune Escape |
|  | IGF1 -> MEF/MYOD/MYOG Expression Targets |
|  | IGF1 -> CEBPA/FOXO1A Expression Targets |
|  | IGF2 -> CEBPA/FOXO1A Expression Targets |
|  | IGF2/MEF/MYOD Expression Targets |
|  | Insulin -> CEBPA/CTNNB/FOXA/FOXO Expression Targets |
|  | Insulin -> MEF/MYOD Expression Targets |
|  | Insulin -> ELK/SRF/HIF1A/MYC/SREBF Expression Targets |
|  | CD19 -> NF-kB Signaling |
|  | AGER -> NF-kB Signaling |
|  | ICAM2 -> CTNNB/FOXO/STAT3 Signaling |
|  | FcIgER -> ELK-SRF Signaling |
|  | InsulinR -> CTNNB/FOXA/FOXO Signaling |
|  | InsulinR -> ELK/SRF/SREBF Signaling |
|  | IGF1R -> CEBPA/FOXO1A Signaling |
|  | IGF1R -> MEF/MYOD/MYOG Signaling |
|  | FibronectinR -> CTNNB Signaling |
|  | FibronectinR -> NF-kB Signaling |
|  | FibronectinR -> AP-1/ELK/SRF/SREBF Signaling |
|  | MacrophageR -> CEBPB -> NF-kB Signaling |
|  | Fibronectin Expression Targets |
|  | CTGF -> AP-1/CREB/MYC Expression Targets |
|  | CTGF -> FOXO3A Expression Target |
|  | CTGF -> NCOR2 Expression Target |
|  | ICAM1 Expression Targets |
|  | Fibrinogen Expression Targets |
|  | IL7R -> FOXO/NF-kB Signaling |
|  | IL15R -> NF-kB/NFATC Signaling |
|  | IL2R -> ELK/SRF/MYC Signaling |
|  | IL23R -> STAT3/NF-kB Signaling |
|  | IL12R -> NF-kB/NFATC Signaling |
|  | IL4R -> ELK/SRF/HMGY Signaling |
|  | IL7 Expression Targets |
|  | IL15 Expression Targets |
|  | IL12B Expression Targets |
|  | IL4 Expression Targets |
|  | IL2 Expression Targets |
|  | UrokinaseR -> ELK/SRF Signaling |
|  | LPA Expression Targets |
|  | PAF/Gq -> AP-1/ATF1/CREB/ERK/SRF Expression Targets |
|  | Dronabinol/Anandamide Expression Targets |
|  | PAF/Gq -> NF-kB Expression Targets |
|  | GPCRs Family -> Expression Targets in Lymphoid System and Blood |
|  | NRG1 -> MEF/MYOD Expression Targets |
|  | NRG1 -> EP300/ETS/ETV/SP1 Expression Targets |
|  | NRG1 -> CREB/CREBBP/ELK/SRF/MYC Expression Targets |
|  | NRG1 -> FOXO3A Expression Targets |
|  | NRG1 -> TP53 Expression Target |
|  | NRG1 -> AP-1/ATF Expression Targets |
|  | NRG1 -> HIF1A Expression Target |
|  | NGF -> SMAD3/NF-kB Expression Targets |
|  | NGF -> AP-1/TP53/MYC Expression Targets |
|  | NGF -> FOXO/MYCN/ELK-SRF Expression Targets |
|  | BDNF Expression Targets |
|  | NTF3 Expression Targets |
|  | NTF4 Expression Targets |
|  | NGF -> CREB/CEBPB/MEF2A Expression Targets |
|  | NOTCH -> NF-kB Signaling |
|  | DLL4 Expression Targets |
|  | ADAM17 Expression Targets |
|  | JAG1 Expression Targets |
|  | DLL1 Expression Targets |
|  | NOTCH Expression Targets |
|  | Adenosine Expression Targets |
|  | PDGFB -> AP-1/CREB/MYC Expression Target |
|  | PDGFB -> FOXO3A Expression Target |
|  | PDGFC -> CREB Expression Target |
|  | PDGFD -> AP-1 Expression Targets |
|  | PDGF -> AP-1/CREB/CREBBP/MYC Expression Targets |
|  | PDGF -> FOXO3A Expression Targets |
|  | KIT -> MITF Signaling |
|  | PDGFR -> AP-1/MYC Signaling |
|  | PDGFR -> FOXO3A Signaling |
|  | AVP/Gs -> CREB/ELK/SRF/AP-1/EGR Expression Targets |
|  | NTS Expression Targets |
|  | AVP/Gs -> MEF/MYOD/NFATC/MYOG Expression Targets |
|  | NPY Expression Targets |
|  | AVP/Gq -> MEF/MYOD/NFATC/MYOG Expression Targets |
|  | AVP/Gq -> CREB/ELK/SRF/AP-1/EGR Expression Targets |
|  | PRL/GHR -> NF/kB/ELK/SRF/MYC Expression Targets |
|  | GUCYC2 Signaling |
|  | AngiopoietinR -> AP-1 Signaling |
|  | AngiopoietinR -> FOXO Signaling |
|  | GDNF -> HSF1 Signaling |
|  | EphrinB -> MAPK/JUN/FOS Signaling |
|  | NTRK -> FOXO/MYCN Signaling |
|  | NTRK -> AP-1/CREB/ELK/SRF/MYC/SMAD3/TP53 Signaling |
|  | GAS6 -> AP-1/CREB Expression Targets |
|  | FIGF -> AP-1 Expression Target |
|  | FIGF -> NCOR2 Expression Target |
|  | KITLG -> AP-1/CREB/CREBBP/MYC Expression Targets |
|  | CSF1 -> FOXO3A Expression Targets |
|  | GDNF -> HSF1 Expression Targets |
|  | CSF1 -> AP-1/CREB/CREBBP/MYC Expression Targets |
|  | PGF -> AP-1/CREB/CREBBP/MYC Expression Targets |
|  | Elevated Receptors -> Expression Targets in Respiratory System |
|  | Lysophosphatidic Acid/LPARs Signaling |
|  | FOXO1 Signaling |
|  | MERTK Signaling |
|  | Ras Signaling |
|  | TGF-beta Signaling |
|  | AXL Receptor Tyrosine Kinase Signaling |
|  | PI3K/RAC1 Signaling |
|  | mTOR Signaling |
|  | TNF-alpha/TNFRSF1B Signaling |
|  | KRAS Signaling |
|  | VEGF Signaling |
|  | Estrogens/ESR1 non-Genomic Signaling |
|  | EGFR Signaling |
|  | HRAS Signaling |
|  | Hedgehog Family Signaling |
|  | T-Cell Receptor -> NF-kB Signaling |
|  | TGFA -> FOXO3A Expression Targets |
|  | TGFA -> TP53 Expression Targets |
|  | TGFA -> HIF1A Expression Targets |
|  | TGFA -> MEF/MYOD/NFATC Expression Targets |
|  | TGFA/AP-1/ATF Expression Targets |
|  | TGFA -> CREB/CREBBP/ELK-SRF/MYC Expression Targets |
|  | CD40LG -> NF-kB/ELK/SRF -> CREB/NFATC Expression Targets |
|  | TNFSF13B Expression Targets |
|  | TNFRSF5/13B -> NFATC1 Signaling |
|  | Thrombopoietin -> SPI1 Expression Targets |
|  | Thrombopoietin -> AP-1/CREB/CREBBP/MYC Expression Targets |
|  | Thrombopoietin -> FOXO3A Expression Target |
|  | TLR1 -> 2/6 Expression Targets |
|  | TLR1/2/6 -> NF-kB Signaling |
|  | Glutamate Mediated Neurotoxicity |
|  | Ca2+ Cytosolic Overload |
|  | ROS in Angiotensin Mediated Cardiovascular Remodeling and Hypertrophy |
|  | PLAU -> ELK-SRF/AP-1 Expression Targets |
|  | VEGFC -> CTNNB Expression Target |
|  | VEGFA -> FOXO3A Expression Targets |
|  | VEGFA -> AP-1/CREBBP/MYC Expression Targets |
|  | VEGFA -> CTNNB/CTNND Expression Targets |
|  | VEGFA -> NCOR2 Expression Target |
|  | VEGFR -> CTNNB Signaling |
|  | VEGFR -> AP-1/CREB/MYC Signaling |
|  | VEGFR -> FOXO3A Signaling |
|  | n |
|  | Model of PPARG signaling in psoriasis |
|  | Model of PPARG related pathways in psoriasis (short version) |
|  | 3\_Increased proliferation of sebocytes in acne vulgaris |
|  | 2\_Interleukin-17 and interleukin-22 signaling in psoriasis |
|  | 1\_The onset of atopic dermatitis |
|  | 2\_Osteoblast-mediated bone demineralization |
|  | 1\_Formation of cysts in polycystic kidney disease |
|  | 3\_Mesangial cell dysfunction in glomerulonephritis |
|  | 1\_Local estrogen production stimulates ectopic endometrium proliferation |
|  | 3\_Enhanced angiogenesis in endometriosis |
|  | 2\_Impaired steroidogenesis in PCOS |
|  | 3\_Role of neurotrophic factor deprivation in retinal ganglion cell death |
|  | 2\_Pathogens stimulate mucins expression in the middle ear |
|  | 3\_Arterial wall inflammation and calcification in atherosclerosis |
|  | 4\_Endothelial cells repress vasoconstriction in hypertension |
|  | 2\_The intense proliferation and contractile activity of smooth muscle cells in pulmonary hypertension |
|  | 1\_Cigarette smoke causes dysfunction and production of inflammatory mediators in alveolar epithelial cells |
|  | 3\_Role of alveolar macrophages in pulmonary tissue destruction in COPD |
|  | 1\_Th2 cell response in asthma |
|  | 3\_1\_Mucus accumulation in asthma |
|  | 2\_2\_Eosinophilia and neutrophilia in asthma: eosinophil apoptosis |
|  | 4\_2\_Airway smooth muscle cells proliferation and airway tissue remodeling |
|  | 4\_Mucin hyperproduction in goblet and mucous cells in COPD |
|  | 2\_Intestine epithelial cells drive immunological responses in ulcerative colitis |
|  | 1\_Triglyceride storage in NAFLD |
|  | 1\_CD4+ T cell and epithelial cell response in celiac disease |
|  | 4\_Tax-induced disruption of the G1/S transition in HTLV-1 infected T-cell |
|  | 2\_HIV - induced depletion of CD4+ T-cells |
|  | 3\_1\_Beta-cell proliferation and compensation: proliferation |
|  | 1\_1\_Insulin synthesis and secretion: insulin processing and storage |
|  | 3\_3\_Beta-cell proliferation and compensation: recovery |
|  | 4\_1\_Insulin resistance: insulin signaling |
|  | 1\_Antigen-presenting cells promote islet dysfunction and immune system activation in T1D |
|  | 1\_Microangiopathy in diabetic neuropathy |
|  | 2\_Neuron and Schwann cell death |
|  | 1\_Glutamate and calcium excitotoxicity of motor neurons in ALS |
|  | 1\_1\_Glutamate overload enforced prostaglandins and NO synthesis in neurons |
|  | 2\_1\_General pathways of thyroid hormone signaling |
|  | Model of PPARG signaling in psoriais (tested) |
|  | DIPG non histone mutations |
|  | DIPG overview\_2 |
|  | HRAS->RAF->MAPK1/3 |
|  | Interleukin-17 and interleukin-22 signaling in psoriasis |

---

|  |  |
| --- | --- |
| Group | Proteins Involved in Cowden Syndrome |

---

|  |  |
| --- | --- |
| Source | curated |
|  | GO component |
|  | Biopax |

---

|  |  |
| --- | --- |
| MedScan ID | 5170 |

---

|  |  |
| --- | --- |
| LocusLink ID | 5170 |
|  | 18607 |
|  | 81745 |
|  | 28993 |
|  | 792239 |

---

|  |  |
| --- | --- |
| Alias | PkB kinase like gene 1 |
|  | 3-phosphoinositide dependent protein kinase I |
|  | OTTMUSP00000022125 |
|  | PDPK1 |
|  | PkB-like |
|  | PkB-like I |
|  | OTTHUMP00000174525 |
|  | PkB kinase |
|  | mPDK1 |
|  | PDPK2 |
|  | PkB-like 1 |
|  | PkB kinase like gene I |
|  | MGC35290 |
|  | MGC20087 |
|  | PDPK I |
|  | PRO0461 |
|  | Phosphoinositide-dependent kinase 1 |
|  | 3' phosphoinositide dependent protein kinase 1 |
|  | Pdk I |
|  | protein kinase B kinase |
|  | hPDK I |
|  | 3-phosphoinositide dependent protein kinase-1 |
|  | PDK1 |
|  | hPDK1 |
|  | mPDK-1 |
|  | OTTMUSP00000022124 |
|  | RP23-153H18.1 |
|  | OTTMUSP00000017452 |
|  | PDPK2P |
|  | 3-phosphoinositide-dependent protein kinase 2 pseudogene |
|  | 3-phosphoinositide-dependent protein kinase 1 |
|  | protein kinase |
|  | PDK-1 |
|  | PIP3 |
|  | PREDICTED: SIMILAR TO 3-PHOSPHOINOSITIDE DEPENDENT PROTEIN KINASE-1(HPDK1). |
|  | scaffold |
|  | chromosome |
|  | hypothetical protein LOC792239 |
|  | LOC792239 |

---

|  |  |
| --- | --- |
| EC Number | 2.7.11.1 |
|  | 2.7.1.37 |

---

|  |  |
| --- | --- |
| GO ID | 0004676 |
|  | 0005524 |
|  | 0005158 |
|  | 0016004 |
|  | 0043274 |
|  | 0019901 |
|  | 0004674 |
|  | 0038095 |
|  | 0031295 |
|  | 0050852 |
|  | 0030036 |
|  | 0032148 |
|  | 0019722 |
|  | 0016477 |
|  | 1990416 |
|  | 0071364 |
|  | 0032869 |
|  | 0007173 |
|  | 0097191 |
|  | 0048041 |
|  | 0006972 |
|  | 0035556 |
|  | 0010667 |
|  | 2000352 |
|  | 0043524 |
|  | 0006469 |
|  | 0034122 |
|  | 0030512 |
|  | 0018105 |
|  | 0018107 |
|  | 0030168 |
|  | 0045766 |
|  | 0043536 |
|  | 0010518 |
|  | 1903078 |
|  | 0051281 |
|  | 1903672 |
|  | 1905564 |
|  | 0046777 |
|  | 0006468 |
|  | 0043122 |
|  | 0043304 |
|  | 0002223 |
|  | 0003323 |
|  | 0042995 |
|  | 0005737 |
|  | 0031410 |
|  | 0005829 |
|  | 0005925 |
|  | 0016020 |
|  | 0005634 |
|  | 0043204 |
|  | 0005886 |
|  | 0014069 |
|  | 0016301 |
|  | 0000166 |
|  | 0004672 |
|  | 0016740 |
|  | 0016310 |
|  | 0010594 |
|  | 0007165 |
|  | 0030054 |
|  | 0048015 |
|  | 0090004 |
|  | 0006355 |
|  | 0006351 |
|  | 0016023 |
|  | 0005622 |
|  | 0097481 |
|  | 0005654 |
|  | 0007596 |
|  | 0008543 |
|  | 0045087 |
|  | 0008286 |
|  | 0048011 |
|  | 0007268 |
|  | 0048010 |
|  | 0016772 |
|  | 0042981 |
|  | 0023034 |
|  | 0007242 |
|  | 0005515 |
|  | 0070201 |
|  | 0004197 |
|  | 0006508 |
|  | 0004713 |
|  | 0000287 |
|  | 0030295 |
|  | 0004682 |
|  | 0004691 |

---

|  |  |
| --- | --- |
| KEGG ID | rno:81745 |
|  | hsa:5170 |
|  | mmu:18607 |

---

|  |  |
| --- | --- |
| MedLine Reference | 10792047 |
|  | 9368760 |
|  | 9637919 |
|  | 9445477 |
|  | 10075713 |
|  | 12783890 |
|  | 14585963 |
|  | 16314505 |
|  | 10349636 |
|  | 11042159 |
|  | 11076861 |
|  | 11217851 |
|  | 11877406 |
|  | 11964154 |
|  | 12006370 |
|  | 12054753 |
|  | 12110585 |
|  | 12177059 |
|  | 12372837 |
|  | 12420226 |
|  | 12466851 |
|  | 12711546 |
|  | 12855688 |
|  | 12912918 |
|  | 12923190 |
|  | 12970179 |
|  | 14500370 |
|  | 14623982 |
|  | 15077109 |
|  | 15116068 |
|  | 15187024 |
|  | 15309345 |
|  | 15389876 |
|  | 15531926 |
|  | 15782199 |
|  | 15961082 |
|  | 16141072 |
|  | 16141073 |
|  | 16150867 |
|  | 16217033 |
|  | 16219676 |
|  | 16243031 |
|  | 16563390 |
|  | 16581799 |
|  | 16642023 |
|  | 16741145 |
|  | 16785309 |
|  | 16825708 |
|  | 16923125 |
|  | 17027005 |
|  | 17077284 |
|  | 8889548 |
|  | 10191262 |
|  | 10226025 |
|  | 10357815 |
|  | 10455013 |
|  | 10480933 |
|  | 10548550 |
|  | 10601311 |
|  | 10698680 |
|  | 10698939 |
|  | 10753910 |
|  | 10764742 |
|  | 10856237 |
|  | 10896934 |
|  | 10945988 |
|  | 10995762 |
|  | 11062054 |
|  | 11222751 |
|  | 11278835 |
|  | 11313365 |
|  | 11376011 |
|  | 11381116 |
|  | 11438723 |
|  | 11445557 |
|  | 11481331 |
|  | 11733037 |
|  | 11779851 |
|  | 11781095 |
|  | 11825911 |
|  | 11889038 |
|  | 12048203 |
|  | 12147684 |
|  | 12149249 |
|  | 12162751 |
|  | 12167717 |
|  | 12169624 |
|  | 12387817 |
|  | 12477932 |
|  | 12485998 |
|  | 12640464 |
|  | 14500673 |
|  | 14604990 |
|  | 14702039 |
|  | 14749367 |
|  | 15059930 |
|  | 15144186 |
|  | 15210700 |
|  | 15302935 |
|  | 15741170 |
|  | 15743829 |
|  | 15802604 |
|  | 15802620 |
|  | 16207722 |
|  | 16288304 |
|  | 9094314 |
|  | 9271440 |
|  | 9373149 |
|  | 9374542 |
|  | 9427642 |
|  | 9430688 |
|  | 9445476 |
|  | 9512493 |
|  | 9748166 |
|  | 9895304 |
|  | 10364160 |
|  | 11157489 |
|  | 15489334 |
|  | 16551362 |
|  | 16807684 |
|  | 17081983 |
|  | 17136479 |
|  | 17255356 |
|  | 8125298 |
|  | 15616553 |
|  | 15554902 |
|  | 16887794 |
|  | 17114649 |
|  | 17599070 |
|  | 17617409 |

---

|  |  |
| --- | --- |
| Organism | Homo sapiens {Organism urn:agi-taxid:9606} |
|  | Mus musculus {Organism urn:agi-taxid:10090} |
|  | Rattus norvegicus {Organism urn:agi-taxid:10116} |
|  | Homo sapiens |
|  | Mus musculus |
|  | Rattus norvegicus |
|  | Danio rerio |
|  | Human |

---

|  |  |
| --- | --- |
| Mouse chromosome position | 17 |
|  | 17 A3.3 |

---

|  |  |
| --- | --- |
| OMIM ID | 605213 |
|  | OMIM:605213 |

---

|  |  |
| --- | --- |
| Rat chromosome position | 10q12 |

---

|  |  |
| --- | --- |
| Hugo ID | 8816 |
|  | HGNC:8816 |

---

|  |  |
| --- | --- |
| Human chromosome position | 16p13.3 |

---

|  |  |
| --- | --- |
| Swiss-Prot Accession | O15530 |
|  | O15530.1 |
|  | Q6A1A2.1 |
|  | Q3TRL2 |
|  | F2Z3X6 |
|  | Q3UGN6 |
|  | Q3UHZ0 |
|  | Q9Z2A0 |
|  | Q9Z2A0.2 |
|  | O55173 |
|  | O55173.2 |
|  | G3V9W3 |
|  | H0Y4Z0 |
|  | Q59EH6 |
|  | Q6FI20 |
|  | Q8IV52 |
|  | Q9BRD5 |
|  | A6H6U3 |
|  | Q9R1D8 |
|  | Q9R215 |
|  | E9PER6 |
|  | Q6A1A2 |
|  | Q53HJ9 |
|  | Q9UPJ7 |
|  | Q9UPJ8 |
|  | Q3UEW8 |
|  | Q810Z4 |
|  | Q8K3L3 |
|  | Q9UI25 |
|  | PDPK1\_HUMAN |
|  | PDPK1\_MOUSE |
|  | PDPK1\_RAT |
|  | Q9BRD5\_HUMAN |
|  | A6H6U3\_MOUSE |
|  | Q3TRL2\_MOUSE |
|  | Q3UHZ0\_MOUSE |

---

|  |  |
| --- | --- |
| GenBank ID | NC\_000016 |
|  | XM\_024450296 |
|  | XP\_024306064 |
|  | XM\_017023274 |
|  | XP\_016878763 |
|  | XM\_017023273 |
|  | XP\_016878762 |
|  | NM\_002613 |
|  | NP\_002604 |
|  | NM\_001261816 |
|  | NP\_001248745 |
|  | XM\_017023277 |
|  | XP\_016878766 |
|  | XM\_017023276 |
|  | XP\_016878765 |
|  | NM\_031268 |
|  | NP\_112558 |
|  | XM\_024450295 |
|  | XP\_024306063 |
|  | XM\_017023275 |
|  | XP\_016878764 |
|  | XM\_017023271 |
|  | XP\_016878760 |
|  | XM\_011522523 |
|  | XP\_011520825 |
|  | XM\_011522521 |
|  | XP\_011520823 |
|  | XM\_017023272 |
|  | XP\_016878761 |
|  | AC005591 |
|  | AAC33797 |
|  | AC093525 |
|  | AC141586 |
|  | AJ406809 |
|  | CAC10873 |
|  | CH471112 |
|  | EAW85495 |
|  | AB209835 |
|  | BAD93072 |
|  | AF017995 |
|  | AAC51825 |
|  | AF111845 |
|  | AAF16685 |
|  | AJ785968 |
|  | CAH05056 |
|  | AK095507 |
|  | AK131060 |
|  | AK222581 |
|  | BAD96301 |
|  | AK293900 |
|  | BAG57287 |
|  | AL157446 |
|  | BC000789 |
|  | BC006339 |
|  | AAH06339 |
|  | BC012103 |
|  | AAH12103 |
|  | BC012540 |
|  | BC033494 |
|  | AAH33494 |
|  | BC040324 |
|  | BI830733 |
|  | CR536517 |
|  | CAG38755 |
|  | Y15056 |
|  | CAA75341 |
|  | O15530 |
|  | Q6A1A2 |
|  | NC\_000083 |
|  | NM\_001286662 |
|  | NP\_001273591 |
|  | NM\_001080773 |
|  | NP\_001074242 |
|  | NM\_011062 |
|  | NP\_035192 |
|  | XM\_017317323 |
|  | XP\_017172812 |
|  | XM\_017317324 |
|  | XP\_017172813 |
|  | XM\_017317325 |
|  | XP\_017172814 |
|  | XM\_017317320 |
|  | XP\_017172809 |
|  | XM\_017317321 |
|  | XP\_017172810 |
|  | XM\_017317322 |
|  | XP\_017172811 |
|  | XM\_017317327 |
|  | XP\_017172816 |
|  | XM\_017317326 |
|  | XP\_017172815 |
|  | AY162410 |
|  | AAO17164 |
|  | CH466606 |
|  | EDL22291 |
|  | CT010502 |
|  | AF079535 |
|  | AAC96115 |
|  | AF086625 |
|  | AAC67544 |
|  | AF126294 |
|  | AAD38505 |
|  | AK047229 |
|  | AK079579 |
|  | AK143283 |
|  | AK147149 |
|  | BAE27716 |
|  | AK147835 |
|  | BAE28171 |
|  | AK149162 |
|  | AK149289 |
|  | BAE28793 |
|  | AK162677 |
|  | BAE37017 |
|  | AK165564 |
|  | AK188106 |
|  | AK193731 |
|  | AK205439 |
|  | AK215448 |
|  | AK219503 |
|  | AY062008 |
|  | AAL47185 |
|  | BC146001 |
|  | AAI46002 |
|  | BC146003 |
|  | AAI46004 |
|  | BQ175596 |
|  | BY257456 |
|  | BY267833 |
|  | CJ046953 |
|  | Q9Z2A0 |
|  | NC\_005109 |
|  | XM\_006246036 |
|  | XP\_006246098 |
|  | NM\_031081 |
|  | NP\_112343 |
|  | AC\_000078 |
|  | AAHX01062886 |
|  | AAHX01062887 |
|  | AAHX01062888 |
|  | AAHX01062889 |
|  | AC130139 |
|  | CH473948 |
|  | EDM03805 |
|  | EDM03806 |
|  | Y15748 |
|  | CAA75758 |
|  | O55173 |
|  | XM\_005255356 |
|  | XP\_005255413 |
|  | XM\_011522524 |
|  | XP\_011520826 |
|  | XM\_011522522 |
|  | XP\_011520824 |
|  | NC\_018927 |
|  | AMYH02031255 |
|  | AMYH02031256 |
|  | AMYH02031257 |
|  | AMYH02031258 |
|  | AMYH02031259 |
|  | AMYH02031260 |
|  | DQ892297 |
|  | ABM83223 |
|  | DQ895496 |
|  | ABM86422 |
|  | EU832784 |
|  | ACE87795 |
|  | GQ129441 |
|  | ACT64575 |
|  | AC\_000039 |
|  | AAHY01131618 |
|  | AAHY01131619 |
|  | AAHY01131620 |
|  | AAHY01131621 |
|  | AAHY01131622 |
|  | XM\_006246038 |
|  | XP\_006246100 |
|  | AC\_000148 |
|  | ABBA01030247 |
|  | ABBA01030248 |
|  | XM\_006523825 |
|  | XP\_006523888 |
|  | XM\_006523828 |
|  | XP\_006523891 |
|  | XM\_006523829 |
|  | XP\_006523892 |
|  | XM\_006523827 |
|  | XP\_006523890 |
|  | XM\_006523826 |
|  | XP\_006523889 |
|  | XM\_006523823 |
|  | XP\_006523886 |
|  | XM\_006523824 |
|  | XP\_006523887 |
|  | XM\_006523830 |
|  | XP\_006523893 |
|  | XM\_006246037 |
|  | XP\_006246099 |
|  | AABR06062999 |
|  | AABR06063000 |
|  | AABR06063001 |
|  | NT\_010393 |
|  | NW\_001838339 |
|  | NW\_926094 |
|  | NW\_927923 |
|  | NW\_926315 |
|  | Q53HJ9 |
|  | Q59EH6 |
|  | Q9BRD5 |
|  | Q9UPJ7 |
|  | Q9UPJ8 |
|  | NT\_039649 |
|  | NW\_001030605 |
|  | Q3TRL2 |
|  | Q3UEW8 |
|  | Q3UGN6 |
|  | Q3UHZ0 |
|  | Q810Z4 |
|  | Q8K3L3 |
|  | NW\_047334 |
|  | NW\_001084656 |
|  | NT\_037887 |
|  | NW\_926018 |
|  | AC\_000059 |
|  | Q6FI20 |
|  | XM\_939834 |
|  | Q9UI25 |
|  | BQ174223 |
|  | NT\_086834 |
|  | NW\_000124 |
|  | XP\_944927 |
|  | 47680172 |
|  | AAC33798 |
|  | 4505695 |
|  | 47680168 |
|  | 47680169 |
|  | 51473214 |
|  | 3513293 |
|  | 3513294 |
|  | 3513295 |
|  | 2407612 |
|  | 2407613 |
|  | 6560611 |
|  | 6560612 |
|  | 21754775 |
|  | 34528313 |
|  | 7018448 |
|  | 13937557 |
|  | 33873510 |
|  | 33873511 |
|  | 15082378 |
|  | 15082379 |
|  | 15214808 |
|  | 23271394 |
|  | 23271395 |
|  | 26007919 |
|  | 15942283 |
|  | 49168519 |
|  | 49168520 |
|  | 2505935 |
|  | 2505936 |
|  | 17380162 |
|  | 9910796 |
|  | 51770238 |
|  | 6755019 |
|  | 6755020 |
|  | 4028126 |
|  | 4028127 |
|  | 3789924 |
|  | 3789925 |
|  | 5052327 |
|  | 5052328 |
|  | 21632794 |
|  | 21632795 |
|  | 18203660 |
|  | 34872015 |
|  | 13592022 |
|  | 13592023 |
|  | 2665355 |
|  | 2665356 |
|  | 18202068 |
|  | NM\_002610 |
|  | 51473210 |
|  | 123784580 |
|  | 123787012 |
|  | 123791192 |
|  | 123791350 |
|  | 28188759 |
|  | 74143425 |
|  | 74151190 |
|  | 74190754 |
|  | 74200494 |
|  | 81895608 |
|  | 81914684 |
|  | 10637404 |
|  | 123997641 |
|  | 62089256 |
|  | 62896721 |
|  | 74721242 |
|  | 74721243 |
|  | 74732868 |
|  | 74740587 |
|  | 74741106 |
|  | 74757560 |
|  | XM\_001130789 |
|  | XP\_001130789 |
|  | NC\_007135 |
|  | NW\_001512718 |
|  | XM\_001331617 |
|  | XP\_001331653 |
|  | 119605901 |
|  | 123982964 |
|  | 123234536 |
|  | 123234537 |
|  | 123234538 |
|  | 123234539 |
|  | 123234543 |
|  | 148690344 |
|  | 148877961 |
|  | 148878393 |
|  | CAM19484 |
|  | CAM19485 |
|  | CAM19486 |
|  | CAM19487 |
|  | CAM19491 |
|  | 149051988 |
|  | 149051989 |
|  | XP\_030105432 |
|  | XP\_030105433 |
|  | XP\_030105434 |
|  | XP\_030105435 |
|  | XM\_030249573 |
|  | XM\_030249572 |
|  | XM\_030249575 |
|  | XM\_030249574 |

---

|  |  |
| --- | --- |
| Swiss-Prot ID | PDPK1\_RAT |
|  | PDPK1\_HUMAN |
|  | PDPK1\_MOUSE |
|  | Q3UHZ0\_MOUSE |
|  | Q3UGN6\_MOUSE |
|  | Q3TRL2\_MOUSE |
|  | Q9BRD5\_HUMAN |
|  | A6H6U3\_MOUSE |

---

|  |  |
| --- | --- |
| Cell Localization | Cytoplasm |
|  | Nucleus |
|  | Cell membrane |
|  | Cell junction |
|  | Focal adhesion |
|  | Cell junction, focal adhesion |
|  | Membrane |
|  | Plasma membrane |

---

|  |  |
| --- | --- |
| Ensembl ID | ENSG00000140992 |
|  | ENSP00000395357.3 |
|  | ENST00000441549.7 |
|  | ENSP00000344220.4 |
|  | ENST00000342085.9 |
|  | ENSP00000268673.7 |
|  | ENST00000268673.11 |
|  | ENSP00000373876.3 |
|  | ENST00000389224.7 |
|  | ENSMUSG00000024122 |
|  | ENSMUSP00000111070.1 |
|  | ENSMUST00000115411.7 |
|  | ENSMUSP00000061942.6 |
|  | ENSMUST00000052462.12 |
|  | ENSMUSP00000099991.3 |
|  | ENSMUST00000102927.9 |
|  | ENSRNOG00000006136 |
|  | ENSRNOP00000061683.1 |
|  | ENSRNOT00000067660.1 |
|  | ENSP00000395357 |
|  | ENST00000441549 |
|  | ENSP00000344220 |
|  | ENST00000342085 |
|  | ENSP00000268673 |
|  | ENST00000268673 |
|  | ENSP00000373876 |
|  | ENST00000389224 |
|  | ENSMUSP00000111070 |
|  | ENSMUST00000115411 |
|  | ENSMUSP00000061942 |
|  | ENSMUST00000052462 |
|  | ENSMUSP00000099991 |
|  | ENSMUST00000102927 |
|  | ENSRNOP00000061683 |
|  | ENSRNOT00000067660 |
|  | ENST00000342085.8 |

---

|  |  |
| --- | --- |
| MGI ID | MGI:1338068 |
|  | 1338068 |

---

|  |  |
| --- | --- |
| RGD ID | 620307 |

---

|  |  |
| --- | --- |
| Unigene ID | Hs.459691 |
|  | Mm.10504 |
|  | Rn.10905 |
|  | Hs.154729 |
|  | Dr.29812 |

---

|  |  |
| --- | --- |
| Homologene ID | 37643 |

---

|  |  |
| --- | --- |
| Shape | Sickle-vertex |

---

|  |  |
| --- | --- |
| IPI ID | IPI00195987 |
|  | IPI00002538 |
|  | IPI00216646 |
|  | IPI00216647 |
|  | IPI00323609 |
|  | IPI00828763 |
|  | IPI00216877 |
|  | IPI00005991 |
|  | IPI00867720 |
|  | IPI00478389 |
|  | IPI00831145 |
|  | IPI00831240 |
|  | IPI00831328 |
|  | IPI00831411 |
|  | IPI00830337 |
|  | IPI00831634 |
|  | IPI00455277 |

---

|  |  |
| --- | --- |
| Chromosome position | 10q12 |
|  | 16p13.3 |
|  | 17 A3.3 |

---

|  |  |
| --- | --- |
| Microarray ID | 1370052\_at |
|  | y15748\_at |
|  | A\_43\_P12504 |
|  | 102063\_at |
|  | 134947\_r\_at |
|  | 1416501\_at |
|  | msa.34345.0\_s\_at |
|  | A\_51\_P207140 |
|  | A\_52\_P619138 |
|  | Y15056 |
|  | 222260\_at |
|  | 32029\_at |
|  | 73168\_s\_at |
|  | 204524\_at |
|  | 224986\_s\_at |
|  | 232050\_at |
|  | RC\_AA041276\_at |
|  | rc\_aa019394\_at |
|  | 221244\_s\_at |
|  | 64052\_at |
|  | rc\_aa488978\_at |
|  | A\_14\_P128757 |
|  | A\_23\_P66219 |
|  | A\_14\_P107472 |
|  | A\_14\_P120883 |
|  | A\_24\_P830690 |
|  | A\_14\_P123854 |
|  | A\_24\_P222599 |
|  | A\_32\_P344843 |
|  | A\_23\_P10614 |
|  | A\_24\_P37441 |
|  | OH8695 |
|  | CA422 |
|  | OH2464 |
|  | OR3282 |
|  | RA1061 |
|  | 160578\_at |

---

|  |  |
| --- | --- |
| Homo sapiens Chromosome position | 16p13.3 |

---

|  |  |
| --- | --- |
| Ensembl Transcript ID | ENST00000268673 |
|  | ENST00000342085 |

---

|  |  |
| --- | --- |
| ENSEMBL | ENSG00000140992 |

---

|  |  |
| --- | --- |
| FunctionalClass | 3-phosphoinositide-dependent protein kinase |
|  | insulin receptor binding |
|  | protein kinase activator |
|  | protein kinase binding |
|  | ATP binding |
|  | kinase |
|  | nucleotide binding |
|  | protein kinase |
|  | protein serine/threonine kinase |
|  | transferase |
|  | protein-tyrosine kinase |
|  | cAMP-dependent protein kinase |
|  | magnesium ion binding |
|  | protein kinase CK2 |
|  | protein binding |

---

|  |  |
| --- | --- |
| Hugo Symbol | PDPK1 |

---

|  |  |
| --- | --- |
| Mus musculus Chromosome position | 17 A3.3 |

---

|  |  |
| --- | --- |
| KEGG pathway | Insulin signaling pathway |
|  | Focal adhesion |
|  | PPAR signaling pathway |
|  | mTOR signaling pathway |
|  | Endometrial cancer |
|  | Non-small cell lung cancer |
|  | Prostate cancer |

---

|  |  |
| --- | --- |
| Rattus norvegicus Chromosome position | 10q12 |

---

|  |  |
| --- | --- |
| Ensembl Gene ID | ENSG00000140992 |

---

|  |  |
| --- | --- |
| ProteinAtlas NormalTissue | Tissue: ovary; Cell type: ovarian stroma cells; Level: Moderate; Expression type: Staining; Reliability: Supportive |
|  | Tissue: appendix; Cell type: lymphoid tissue; Level: Moderate; Expression type: Staining; Reliability: Supportive |
|  | Tissue: cervix |
|  | uterine; Cell type: squamous epithelial cells; Level: Weak; Expression type: Staining; Reliability: Supportive |
|  | Tissue: stomach |
|  | upper; Cell type: glandular cells; Level: Moderate; Expression type: Staining; Reliability: Supportive |
|  | Tissue: vulva/anal skin; Cell type: epidermal cells; Level: Moderate; Expression type: Staining; Reliability: Supportive |
|  | Tissue: vagina; Cell type: squamous epithelial cells; Level: Moderate; Expression type: Staining; Reliability: Supportive |
|  | Tissue: cerebellum; Cell type: cells in granular layer; Level: Negative; Expression type: Staining; Reliability: Supportive |
|  | Tissue: uterus |
|  | post-menopause; Cell type: glandular cells; Level: Moderate; Expression type: Staining; Reliability: Supportive |
|  | pre-menopause; Cell type: glandular cells; Level: Moderate; Expression type: Staining; Reliability: Supportive |
|  | Tissue: adrenal gland; Cell type: glandular cells; Level: Moderate; Expression type: Staining; Reliability: Supportive |
|  | Tissue: appendix; Cell type: glandular cells; Level: Moderate; Expression type: Staining; Reliability: Supportive |
|  | Tissue: bone marrow; Cell type: hematopoietic cells; Level: Moderate; Expression type: Staining; Reliability: Supportive |
|  | Tissue: breast; Cell type: glandular cells; Level: Moderate; Expression type: Staining; Reliability: Supportive |
|  | Tissue: bronchus; Cell type: respiratory epithelial cells; Level: Moderate; Expression type: Staining; Reliability: Supportive |
|  | Tissue: cerebellum; Cell type: cells in molecular layer; Level: Moderate; Expression type: Staining; Reliability: Supportive |
|  | Tissue: cerebellum; Cell type: Purkinje cells; Level: Moderate; Expression type: Staining; Reliability: Supportive |
|  | Tissue: cerebral cortex; Cell type: glial cells; Level: Weak; Expression type: Staining; Reliability: Supportive |
|  | Tissue: cerebral cortex; Cell type: neuronal cells; Level: Moderate; Expression type: Staining; Reliability: Supportive |
|  | Tissue: cervix, uterine; Cell type: glandular cells; Level: Moderate; Expression type: Staining; Reliability: Supportive |
|  | Tissue: cervix, uterine; Cell type: squamous epithelial cells; Level: Weak; Expression type: Staining; Reliability: Supportive |
|  | Tissue: colon; Cell type: glandular cells; Level: Moderate; Expression type: Staining; Reliability: Supportive |
|  | Tissue: duodenum; Cell type: glandular cells; Level: Strong; Expression type: Staining; Reliability: Supportive |
|  | Tissue: epididymis; Cell type: glandular cells; Level: Moderate; Expression type: Staining; Reliability: Supportive |
|  | Tissue: esophagus; Cell type: squamous epithelial cells; Level: Weak; Expression type: Staining; Reliability: Supportive |
|  | Tissue: fallopian tube; Cell type: glandular cells; Level: Moderate; Expression type: Staining; Reliability: Supportive |
|  | Tissue: gall bladder; Cell type: glandular cells; Level: Moderate; Expression type: Staining; Reliability: Supportive |
|  | Tissue: heart muscle; Cell type: myocytes; Level: Negative; Expression type: Staining; Reliability: Supportive |
|  | Tissue: hippocampus; Cell type: glial cells; Level: Weak; Expression type: Staining; Reliability: Supportive |
|  | Tissue: hippocampus; Cell type: neuronal cells; Level: Moderate; Expression type: Staining; Reliability: Supportive |
|  | Tissue: kidney; Cell type: cells in glomeruli; Level: Moderate; Expression type: Staining; Reliability: Supportive |
|  | Tissue: kidney; Cell type: cells in tubules; Level: Moderate; Expression type: Staining; Reliability: Supportive |
|  | Tissue: lateral ventricle; Cell type: glial cells; Level: Moderate; Expression type: Staining; Reliability: Supportive |
|  | Tissue: lateral ventricle; Cell type: neuronal cells; Level: Moderate; Expression type: Staining; Reliability: Supportive |
|  | Tissue: liver; Cell type: bile duct cells; Level: Negative; Expression type: Staining; Reliability: Supportive |
|  | Tissue: liver; Cell type: hepatocytes; Level: Negative; Expression type: Staining; Reliability: Supportive |
|  | Tissue: lung; Cell type: macrophages; Level: Moderate; Expression type: Staining; Reliability: Supportive |
|  | Tissue: lung; Cell type: pneumocytes; Level: Weak; Expression type: Staining; Reliability: Supportive |
|  | Tissue: lymph node; Cell type: germinal center cells; Level: Moderate; Expression type: Staining; Reliability: Supportive |
|  | Tissue: lymph node; Cell type: non-germinal center cells; Level: Moderate; Expression type: Staining; Reliability: Supportive |
|  | Tissue: nasopharynx; Cell type: respiratory epithelial cells; Level: Moderate; Expression type: Staining; Reliability: Supportive |
|  | Tissue: oral mucosa; Cell type: squamous epithelial cells; Level: Weak; Expression type: Staining; Reliability: Supportive |
|  | Tissue: pancreas; Cell type: exocrine glandular cells; Level: Moderate; Expression type: Staining; Reliability: Supportive |
|  | Tissue: pancreas; Cell type: islets of Langerhans; Level: Moderate; Expression type: Staining; Reliability: Supportive |
|  | Tissue: parathyroid gland; Cell type: glandular cells; Level: Moderate; Expression type: Staining; Reliability: Supportive |
|  | Tissue: placenta; Cell type: decidual cells; Level: Moderate; Expression type: Staining; Reliability: Supportive |
|  | Tissue: placenta; Cell type: trophoblastic cells; Level: Strong; Expression type: Staining; Reliability: Supportive |
|  | Tissue: prostate; Cell type: glandular cells; Level: Moderate; Expression type: Staining; Reliability: Supportive |
|  | Tissue: rectum; Cell type: glandular cells; Level: Moderate; Expression type: Staining; Reliability: Supportive |
|  | Tissue: salivary gland; Cell type: glandular cells; Level: Moderate; Expression type: Staining; Reliability: Supportive |
|  | Tissue: seminal vesicle; Cell type: glandular cells; Level: Moderate; Expression type: Staining; Reliability: Supportive |
|  | Tissue: skeletal muscle; Cell type: myocytes; Level: Negative; Expression type: Staining; Reliability: Supportive |
|  | Tissue: skin; Cell type: epidermal cells; Level: Negative; Expression type: Staining; Reliability: Supportive |
|  | Tissue: small intestine; Cell type: glandular cells; Level: Moderate; Expression type: Staining; Reliability: Supportive |
|  | Tissue: smooth muscle; Cell type: smooth muscle cells; Level: Weak; Expression type: Staining; Reliability: Supportive |
|  | Tissue: spleen; Cell type: cells in red pulp; Level: Negative; Expression type: Staining; Reliability: Supportive |
|  | Tissue: spleen; Cell type: cells in white pulp; Level: Moderate; Expression type: Staining; Reliability: Supportive |
|  | Tissue: stomach, lower; Cell type: glandular cells; Level: Moderate; Expression type: Staining; Reliability: Supportive |
|  | Tissue: stomach, upper; Cell type: glandular cells; Level: Moderate; Expression type: Staining; Reliability: Supportive |
|  | Tissue: testis; Cell type: cells in seminiferus ducts; Level: Strong; Expression type: Staining; Reliability: Supportive |
|  | Tissue: testis; Cell type: Leydig cells; Level: Moderate; Expression type: Staining; Reliability: Supportive |
|  | Tissue: thyroid gland; Cell type: glandular cells; Level: Strong; Expression type: Staining; Reliability: Supportive |
|  | Tissue: tonsil; Cell type: germinal center cells; Level: Moderate; Expression type: Staining; Reliability: Supportive |
|  | Tissue: tonsil; Cell type: non-germinal center cells; Level: Moderate; Expression type: Staining; Reliability: Supportive |
|  | Tissue: tonsil; Cell type: squamous epithelial cells; Level: Moderate; Expression type: Staining; Reliability: Supportive |
|  | Tissue: urinary bladder; Cell type: urothelial cells; Level: Moderate; Expression type: Staining; Reliability: Supportive |
|  | Tissue: uterus, post-menopause; Cell type: cells in endometrial stroma; Level: Moderate; Expression type: Staining; Reliability: Supportive |
|  | Tissue: uterus, post-menopause; Cell type: glandular cells; Level: Moderate; Expression type: Staining; Reliability: Supportive |
|  | Tissue: uterus, pre-menopause; Cell type: cells in endometrial stroma; Level: Weak; Expression type: Staining; Reliability: Supportive |
|  | Tissue: uterus, pre-menopause; Cell type: glandular cells; Level: Moderate; Expression type: Staining; Reliability: Supportive |

---

|  |  |
| --- | --- |
| EnzymeConsortium | 2.7.1.37 |

---
